# The case for consistent use of medical eponyms by eliminating possessive forms

**DOI:** 10.5195/jmla.2018.284

**Published:** 2018-01-02

**Authors:** Kwabena Ayesu, Brenda Nguyen, Stephanie Harris, Steve Carlan

The use of medical eponyms, which are medical terms named after people (e.g., Down’s syndrome), has frequently been a source of confusion for learners. Although the US National Institutes of Health (NIH) and the World Health Organization have encouraged the medical community to refrain from the use of possessive eponyms in order to reduce the misconception that the disease founders suffered from the pathology themselves, the inconsistent use of possessive and non-possessive forms of medical eponyms is still common.

The objective of this commentary is to highlight the pervasive usage of both forms of medical eponyms in medical literature amongst prestigious medical journals indexed in the PubMed database. This use of eponyms poses a source of confusion in literature searching as well as a lack of consistency in medical education. The adoption of consistent non-possessive forms should be encouraged by editors, reviewers, and publishers.

## INTRODUCTION

Since 1974, NIH has recommended refraining from using possessive eponyms [1], which are medical terms named after people that include an “s” at the end. For example, trisomy-21 is a genetic condition that describes a person who was born with an extra 21st chromosome and who has classic clinical findings. At the time of the official description of the disease in 1866, neither genetic studies nor an understanding of the fundamentals of genetic disease expression were available, therefore the condition was named after the investigator, John Down, and subsequently became known as Down’s syndrome.

Unfortunately, despite criticisms, the possessive forms of medical eponyms remain in common use (e.g., Down’s syndrome rather than Down syndrome). Inconsistency in the use of eponyms in medical literature poses a problem not only to scholarly writing, but also to medical education [2]. Whereas the possessive form was applied nearly universally to medical literature from 1960s until the early 1970s [3], arguments both for and against the use of possessive medical eponyms emerged in the mid-1970s. Since then, however, there has been a slow move away from the use of possessive medical eponyms. “Stigler’s law of eponymy” from 1980 stated that no scientific discovery should be named after its original discoverer [3]. In addition, the World Health Organization in 2004 and the American Medical Association in 2007 advocated for eliminating the possessive form [4, 5]. This commentary highlights the dilemma of continued use of possessive forms of eponyms in prestigious journals indexed in the PubMed database.

## MEDICAL EPONYMOUS QUERY ENTRY

Novice searchers and those without formal training may find a search for comprehensive disease-specific literature to be inconsistent and confusing, based on whether a medical eponym is searched in the possessive or non-possessive form. While a search using PubMed’s controlled vocabulary—Medical Subject Headings (MeSH)—retrieves relevant articles, this technique retrieves only those articles that are formally indexed.

When PubMed is filtered to the “Core Clinical Journals” subset and medical queries are searched, the number of returned articles significantly differs depending on the form of the query entered. For example, the authors searched for common medical eponymous diseases based on reports generated by NIH and Harvard Health Publications in PubMed. The specific search queries and the numbers of returned articles are shown in Table 1.

**Table 1:** Results of PubMed search queries for medical eponyms

Medical query	Manuscripts generated	Manuscripts omitted	Total manuscripts
Alzheimer’s disease	8,270	0	—
Alzheimer disease	7,405	865	—
Alzheimer’s OR Alzheimer disease	8,270	0	8,270
Crohn’s disease	7,419	0	—
Crohn disease	6,506	913	—
Crohn’s disease OR Crohn disease	7,419	0	7,419
Grave’s disease	2,828	186	—
Grave disease	188	2,826	—
Grave’s disease OR Grave disease	3014	0	3,014
Non-Hodgkin’s lymphoma	13,221	227	—
Non-Hodgkin lymphoma	12,729	719	—
Non-Hodgkin’s lymphoma OR Non-Hodgkin lymphoma	13,448	0	13,448
Parkinson’s disease	7,683	0	—
Parkinson disease	7,180	503	—
Parkinson’s disease OR Parkinson disease	7,683	0	7,683

As the data demonstrate, although the non-possessive form is the currently advocated proper form, a substantial number of articles were omitted when non-possessive forms of eponyms were searched between the dates of January 2015 and January 2017. This omission was observed for non-possessive search entries for Alzheimer disease, Crohn disease, and Parkinson disease, which omitted 865, 913, and 503 articles, respectively, whereas no articles were missed when the possessive forms were entered. By contrast, a search for non-Hodgkin’s disease, the possessive form, omitted over 200 manuscripts, whereas the non-possessive form omitted 719 publications. Although a total of 13,448 articles are currently available on PubMed for non-Hodgkin’s and non-Hodgkin disease, these articles are not fully retrieved unless an expansive search algorithm is used, which is not commonly understood by novice searchers.

Grave disease, on the other hand, poses a special dilemma because when the query is entered as the non-possessive form, the eponym “Grave” can be misinterpreted as a descriptive term by the database to mean a grave (i.e., serious) disease instead of the actual pathology. The entry for “Grave disease” omitted over 2,000 manuscripts, whereas use of the possessive eponym returned 2,828 articles. This difference poses a separate argument regarding the use of medical eponyms versus scientific pathologic description, which will not be discussed here.

## CONCLUSION

This initiative is not aimed at removing diversity in medical literature, but rather at reducing a possible source of confusion. Implementing these changes will bring about a more consistent and efficient method of literature searching. Over time, the possessive forms of eponyms have seen a slow and gradual decline as many major international organizations recognize that the non-possessive form is more efficient, simpler, and not confusing.

Mathematical models show that changes in social consensus can be attained through various sizes of organizations that hold influential roles in medical society [3]. Therefore, the first steps to eliminating possessive eponyms would require an organized effort. Awareness and change toward implementing and using appropriate medical nomenclature is the responsibility not only of authors, but also of editors, reviewers, and publishers.

Our data show that literature searches with possessive eponymous queries are often able to yield all available articles in the database, whereas searches for the non-possessive form often omit a large quantity of articles. Awareness and efforts should be made to standardize eponyms to enhance literature searching and to adopt the non-possessive form exclusively in future works that are published.
